# The association between acne care provision and quality of life: A cross‐sectional survey

**DOI:** 10.1002/hsr2.487

**Published:** 2022-02-08

**Authors:** Femke de Vries, Rieke Driessen, Esther Tjin, Anissa Westenberg, Hans Vehof, Peter van de Kerkhof

**Affiliations:** ^1^ Research Group Innovations in Pharmaceutical Care HU University of Applied Sciences Utrecht Utrecht The Netherlands; ^2^ Department of Dermatology Radboud University Medical Center Nijmegen The Netherlands

**Keywords:** acne vulgaris, quality of life, treatment

## Abstract

**Background and Aims:**

It has been suggested that professional acne care can be effective not only in reducing clinical signs but also in improving quality of life (QOL). This study aims to reach a better understanding of the association between QOL and professional acne care. The study also investigates other factors that might influence QOL such as age, gender, and acne severity.

**Methods:**

Between 2019 and 2020, a cross‐sectional survey‐based study was conducted among 362 acne patients. Data were collected by the Cardiff Acne Disability Index (CADI) and a Global QOL scale. Analysis of covariance (ANCOVA) and post hoc comparisons were conducted to analyze the association between professional acne care and health‐related QOL.

**Results:**

No statistically significant differences were found in QOL measured by CADI among patients visiting the four investigated acne caregivers (mean CADI score: dermatologist, 4.49; GPs, 4.42; dermal therapist, 4.07; beautician, 4.20, *P* = .24). However, the impact of the treatment on the QOL, which was measured by the level of Global QOL improvement before and after care, demonstrated a statistically significant improvement when attending a dermatologist, compared to the care provided by beauticians (Global QOL improvement: dermatologist, 1.50; GP, 1.01; dermal therapist, 1.10; beautician, 0.54, *P* = .05). Females experienced a more impaired acne‐related QOL than males (*P* = .05), and increased acne severity was associated with a more impaired QOL (*P* < .05).

**Conclusions:**

This study delineated factors that influence QOL in acne patients. As the QOL was not associated with the type of caregiver, the greatest QOL improvement before and after care was achieved after medical treatment by the dermatologist. Females and individuals dealing with more severe types of acne experienced a more impaired acne‐related QOL. It is recommended to take these factors into account in acne management to optimize professional treatment in line with patient needs.

## 1 INTRODUCTION

Acne vulgaris is one of the most common skin disorders, affecting more than 85% of individuals at least once in their lives. While acne is most prevalent among adolescents aged between 15 and 24 years, it is not uncommon in adults.^1^, ^2^ Acne is defined as a chronic inflammatory dermatosis notable for comedones and inflammatory lesions, including papules, pustules and nodules, and can progress into scars, postinflammatory hyperpigmentation or both.^3^ Acne most commonly affects the visible skin, a vital organ of social display, and peaks in teenage years, which can be considered a pivotal time for physical, emotional, and social development. As acne is often considered a cosmetic and non‐life‐threatening condition, the consequences are frequently underestimated by medical practitioners. However, acne can have long‐term consequences, a profound negative psychological impact, and implications for patients' quality of life (QOL). Previous studies have demonstrated that acne is associated with the presence of psychological problems such as anxiety, depression, feelings of uselessness, fewer feelings of pride, social isolation, aggression, frustration, problems with social activities, and personal relationships and lower body satisfaction.^4^, ^5^, ^6^, ^7^, ^8^, ^9^ Studies have also indicated that the negative psychological impact of having acne is comparable to that of chronic medical conditions, including asthma, epilepsy, diabetes mellitus, back pain, and arthritis.^4^, ^10^ Despite the negative impact of acne on QOL, not all individuals with acne experience the same level of QOL impairment, indicating that other factors might be at work.^11^, ^12^, ^13^, ^14^

Many factors that influence QOL have been investigated before with contradictory results; for example, age, gender, and acne severity. Another factor that might influence QOL in individuals with acne is receiving professional care.^12^ It has been shown that professional care can be effective, not only in reducing the clinical signs but also by affecting the psychological well‐being and QOL.^11^, ^13^, ^15^, ^16^ In the Netherlands, professional acne care is utilized by four main types of acne caregivers: dermatologists and general practitioners (GPs), mainly applying pharmacological treatment modalities. Non‐physician caregivers, including dermal therapists—medical skin care professionals with bachelor's degrees—and beauticians, mainly provide nonpharmacological treatment modalities, that is, light and laser‐based treatments, chemical peels, and lesion removal. Despite the variety of Dutch acne care services and caregivers, the impact of professional acne care on the QOL remains unclear. Identifying the possible value of care(givers) on the acne‐related QOL may contribute to adopting QOL measures as an integral part of acne management. This may improve the quality of acne care provision in clinical practice that is more in line with the patients' needs.^17^ The primary objective of this study was to investigate the association between professional care and the QOL, with particular emphasis on the various types of acne caregivers in the Netherlands.

## 2 MATERIALS AND METHODS

### 2.1 Study design

To assess the impact of acne vulgaris on health‐related QOL, a cross‐sectional, questionnaire‐based study was conducted involving Dutch individuals with acne. The study was reported according to the recommendations in the Strengthening the Reporting of Observational Studies in Epidemiology checklist.^18^

### 2.2 Setting

Recruitment of subjects took place between March and September 2019 in The Netherlands. Eligibility for participation was restricted to those who spoke and read Dutch, were at least 18 years of age, were suffering from or recently recovered from (self‐diagnosed) acne vulgaris, preferably had sought professional help from an acne care provider, were either male or female, and were of any Fitzpatrick skin type. Subjects were recruited through posters and flyers that were distributed in vocational schools (Dutch: MBOs), higher vocational schools (Dutch: HBOs), universities (Dutch: WOs), and healthcare organization waiting rooms across the country. Subjects were also recruited via several social media platforms. All recruitment channels contained a URL link and a QR code that directly corresponded to a digital questionnaire. Data were collected using the survey platform LimeSurvey^19^ and subsequently transported to the Statistical Package for Social Science (SPSS) version 25 for further analysis. A sample size was calculated on a minimum of 384 respondents with 95% confidence interval (CI) and 5% error margin.

### 2.3 Outcomes

To determine the impact of acne on QOL, the CADI questionnaire was used. The CADI is a validated acne‐specific health‐related QOL questionnaire containing five questions about feelings, social life, skin exposure, and overall severity. The responses to each question are on a 4‐point scale. The CADI score was calculated by summing the score of each question (0‐3), resulting in a minimum score of 0 and a maximum score of 15. Higher scores indicate a more severely impaired QOL. The data of respondents with more than one incomplete answer on the CADI questions were excluded from the study.^4^, ^20^ To assess the self‐reported level of QOL improvement before and after care, two self‐developed, nonvalidated health‐related Global QOL questions were used. First, we retrospectively asked about the past QOL: “What was your (acne‐related) QOL before seeking any kind of professional treatment?” Second, we asked about the present QOL “What is your (acne‐related) QOL after seeking any kind of professional treatment?” Response options ranged from 0 to 10 (0 = acne does not affect QOL, 10 = acne strongly affects QOL). We subtracted the “before” Global QOL response‐score from the “after” Global QOL response‐score from each respondent, to assess an individual level of improvement in QOL. To assess acne severity, respondents were requested to compare their own acne severity before and after care to one of the five displayed photographs (0 = clear, 4 = severe) based on the Investigator's Global Assessment (IGA) (Canfield), further mentioned as Patient Global Assessment (PtGA).^21^, ^22^ Finally, respondents were asked whether they had received professional care and, if so, were requested to report the type of caregiver. For respondents who had attended more than one caregiver, the last caregiver in the care pathway was used for further analysis.

### 2.4 Data analysis

Data were analyzed using SPSS version 25 (SPSS Inc., Chicago, IL, USA). Descriptive statistics including frequency, mean, range, percentage, and SD were calculated to describe the respondents' characteristics. A one‐way analysis of variance (ANOVA) and Bonferroni post hoc test were used to analyze the differences between the mean CADI score and the PtGA acne severity score. Furthermore, analyses of covariance (ANCOVA) and Bonferroni post hoc test were used to analyze the QOL in acne patients visiting the different types of caregivers, which was captured with (a) CADI and (b) Global QOL improvement before and after visited caregiver. Factors that derived from literature and a pre‐analysis (Spearman correlation) that could potentially influence the QOL were acne severity, age, and gender. These factors were taken into account as covariants within all analyses. A *P*‐value ≤.05 was considered statistically significant.

### 2.5 Ethics

The study protocol was approved by the Medical Ethics Committee of the Radboud University Medical Center, Nijmegen (registration number 2018‐5005), which declared that the study did not fall under the scope of the “Medical Research Involving Human Subjects Act”. The digital questionnaire was preceded by a description of the study rationale and informed consent form, which must have been read and accepted before beginning the actual questionnaire. Furthermore, the anonymity and confidentiality of subjects were guaranteed throughout the study by replacing directly identifiable personal data with questionnaire numbers.

## 3 RESULTS

### 3.1 Demographics and acne characteristics

A total of 371 CADI questionnaires were completed. Nine were excluded due to having more than one incomplete answer, leaving 362 questionnaires for analysis. The study population consisted of 79% females and 21% males. Most respondents were aged between 21 and 30 years (64.9%). Before receiving any type of care, most respondents evaluated their acne as PtGA 3 (49%%). After receiving care, most respondents evaluated their acne as PtGA 1 (37.4%). The mean age ± SD of the onset of acne was 14.43 ± 3.80 years. The respondents' demographics and acne characteristics are outlined in Table 1.

**TABLE 1:** Demographics and acne characteristics

	N	%
Age		
≤20	63	17.4
21‐30	235	64.9
31‐40	45	12.4
≥41	19	5.3
Sex		
Female	282	79
Male	77	21
Type of caregiver		
Beautician	66	23.1
Dermal therapist	75	26.2
GP	67	23.4
Dermatologist	70	24.5
Acne severity PtGA before care		
0	1	0.3
1	18	6.3
2	75	26.2
3	140	49
4	52	18.2
Acne severity PtGA after care		
0	65	22.7
1	107	37.4
2	76	26.6
3	32	11.2
4	6	2.1
Mean age acne onset ± SD	14.4 ± 3.80	
Duration of acne		
<1 y	25	6.9
1‐5 y	131	36.2
5‐10 y	127	35.1
>10 y	79	21.8

Abbreviations: PtGA, Patient Global Assessment; SD, standard deviation.

### 3.2 The association between QOL and acne severity

**FIGURE 1:**
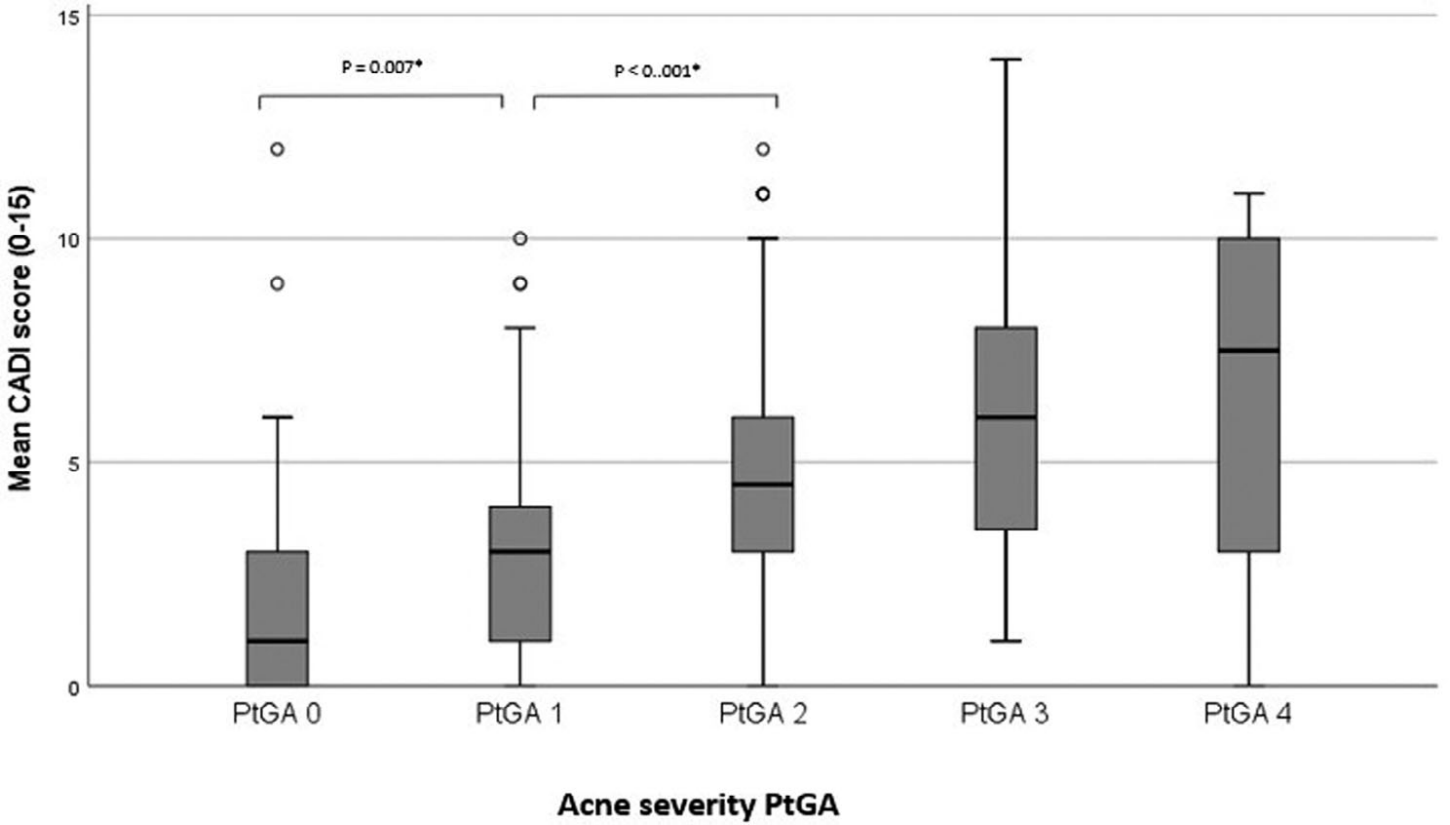
Box plot and post hoc comparison of PtGA acne severity by CADI score. *P‐value ≤.05 indicates statistically significant differences between acne severity PtGA scores

For each PtGA score, the mean ± SD QOL, as measured by the CADI, is as follows: PtGA 0, 1.71 ± 2.47; PtGA 1, 2.88 ± 2.15; PtGA 2, 4.70 ± 2.64; PtGA 3, 6.00 ± 3.09; and PtGA 4, 5.86 ± 4.30. Furthermore, an increase in acne severity was associated with a more impaired QOL (F[4, 357] = 28 832, *P* < .001). A post hoc analysis demonstrated statistically significant differences between the lower acne severity PtGA scores 0, 1, and 2 (Figure 1).

### 3.3 The association between QOL and type of acne caregiver

**FIGURE 2:**
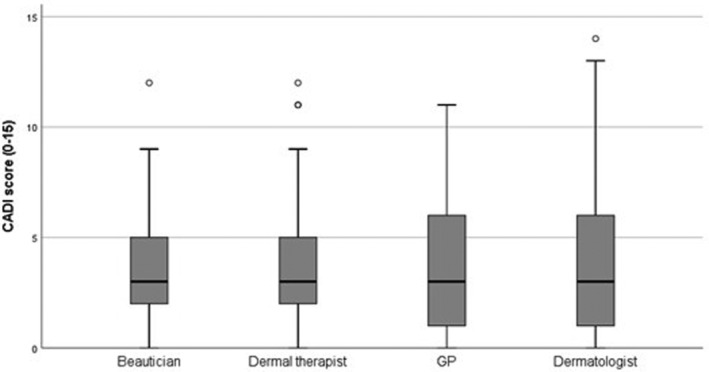
Box plot for mean CADI score by caregiver attended

The association between QOL, as measured by CADI, and the type of caregiver was investigated using an ANCOVA. Small but not statistically significant differences were found in the QOL of patients visiting one of the four investigated acne caregivers, indicating that the type of caregiver did not affect the QOL (F[3266] 1.409 *P* = .24). Mean CADI score; dermatologist, 4.49; GPs, 4.42; dermal therapist, 4.07; beautician, 4.20, Table 2, Figure 2. However, gender and acne severity were factors directly affecting the QOL in the patients with acne, finding that females experienced a more impaired acne‐related QOL than males (females, 4.82; males 4.00, *P* = .05) and increased acne severity was associated with a more impaired QOL (PtGA 0, 1.70; PtGA 1, 2.91; PtGA 2, 4.72; PtGA 3, 5.68; PtGA 4, 7.04, *P* < .01).

**TABLE 2:** ANCOVA mean CADI scores by caregiver attended, unadjusted, and adjusted for gender, age, and acne severity

	Model 1 (unadjusted)					Model 2 (adjusted)				
	Mean CADI		95% CI		*P*	Mean CADI		95% CI		*P*
		SE	Lower	Upper			SE	Lower	Upper	
Type of caregiver					.74					.24
Beautician	3.47	0.37	2.74	4.20		4.20	0.43	3.35	5.05	
Dermal therapist	3.68	0.35	2.99	4.37		4.07	0.42	3.24	4.90	
GP	3.78	0.37	3.05	4.50		4.42	0.40	3.63	5.21	
Dermatologist	4.04	0.36	3.33	4.75		4.95	0.42	4.13	5.77	
Gender										.05*
Female						4.82	0.29	4.26	5.39	
Male						4.00	0.45	3.11	4.88	
Age^a^						−0.02	0.02	−0.06	0.02	.28
Acne severity^b^										<.001*
0						1.70	0.36	0.99	2.41	
1						2.91	0.30	2.32	3.50	
2						4.72	0.35	4.03	5.41	
3						5.68	0.50	4.70	6.66	
4^b^						7.04	1.20	4.69	9.40	

^a^ Unstandardized B was reported for age.

^b^ Acne severity PtGA 0‐4.

* *P*‐value ≤.05 indicates statistically significant association with QOL.

### 3.4 Level of improvement in Global QOL before and after care

**FIGURE 3:**
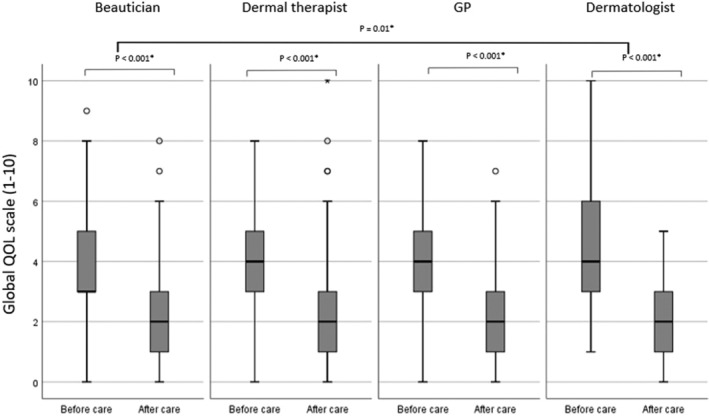
Box plot Global QOL before and after care by caregiver attended. *P‐value ≤.05 indicates statistically significant difference in the level of improvement before and after attending caregiver and between dermatologist and beautician

An additional ANCOVA using the self‐reported differences in Global QOL provided valuable information regarding the impact of the treatment by the level of QOL improvement before and after care, which was investigated between different combinations of caregivers. The greatest improvement was found after medical treatment by the dermatologist, which differed significantly compared to the care provided by beauticians (F[3266] = 2.571, *P* = .05). Mean level of improvement in Global QOL before and after care; dermatologist, 1.50; GP, 1.01; dermal therapist, 1.10; beautician, 0.54, *P* = .05; Table 3, Figure 3. Moreover, the factors gender and acne severity directly affected the level of QOL improvement in patients with acne, finding that females experienced a higher level of improvement in QOL than males (females, 1.38; males, 0.69, *P* = .03) and patients with higher acne severity rates experienced less improvement in QOL (PtGA 0, 2.41; PtGA 1, 1.75; PtGA 2, 0.85; PtGA 3, 0.70; PtGA 4, −0.53, *P* < .001.

**TABLE 3:** ANCOVA mean level of improvement in Global QOL before and after attending caregiver, unadjusted, and adjusted for gender, age, and acne severity

	Model 1 (unadjusted)					Model 2 (adjusted)				
	Mean improvement	SE	95% CI		*P*	Mean improvement	SE	95% CI		*P*
			Lower	Upper				Lower	Upper	
Type of caregiver^a^					<.05*					.05*
Beautician	1.29	0.26	0.78	1.79		0.54	0.32	−0.09	1.18	
Dermal therapist	1.77	0.24	1.30	2.25		1.10	0.32	0.48	1.72	
GP	1.61	0.25	1.11	2.11		1.01	0.30	0.41	1.60	
Dermatologist	2.27	2.27	1.78	2.76		1.50	0.31	0.88	2.11	
Gender										.03*
Female						1.38	0.22	0.96	1.81	
Male						0.69	0.34	0.03	1.35	
Age^b^						0.00	0.02	−0.03	0.03	.88
Acne severity^c^										<.001*
0						2.41	0.27	1.88	2.95	
1						1.75	0.22	1.31	2.19	
2						0.85	0.26	0.33	1.36	
3						0.70	0.37	−0.04	1.43	
4						−0.53	0.90	−2.29	1.23	

^a^ Bonferroni post hoc demonstrates statistically significant difference in the level of improvement between dermatologist and beautician.

^b^ Unstandardized B was reported for age.

^c^ Acne severity PtGA 0‐4.

* *P*‐value ≤.05 indicates statistically significant association with QOL.

## 4 DISCUSSION

This study delineated factors that influence QOL in individuals with acne. The type of caregiver visited did not influence the QOL. However, the QOL before and after professional care, which was investigated between different combinations of caregivers, demonstrated a statistically significant improvement when attending a dermatologist, compared to the care provided by beauticians. Moreover, female gender and an increase in acne severity were associated with a more impaired acne‐related QOL.

Discrepancies in the QOL improvement between dermatologists and beauticians visited might be explained by the fact that patients visiting a beautician initially experienced a milder sense of QOL impairment, compared to patients receiving medical treatments from dermatologists. In the Netherlands, beauticians are often considered an easy and approachable first step toward acne treatments. Dermatologists, on the other hand, are often a last step into the care chain and are exclusively accessible with a referral letter provided by a GP. This may have caused a delay between the onset of acne and a dermatological treatment, reinforcing the concept of stigmatization or remaining clinical signs of persistent acne (such as scars or postinflammatory hyperpigmentation), influencing the QOL more negatively to begin with References 23, 24, 25. It is also plausible that our findings were caused by higher levels of acne severity which are more often seen by dermatologists, simultaneously dealing with patients having more impaired QOL. However, we were not able to detect such, meaning acne severity was not associated with the decision to visit a particular type of caregiver. This was also observed in a previous study, finding no statistically significant association between the type of caregiver with respect to the disease severity.^26^ Finally, a positive experience with a particular type of caregiver may have influenced our findings, that is, having a positive experience with an effective dermatological treatment will presumably enhance the patients' QOL.^27^, ^28^

Furthermore, our study revealed that QOL is not solely affected by the type of professional care; other factors play a role. First, we found that gender influences QOL in a highly significant way. In our study, female respondents tended to experience a more impaired acne‐related QOL than male respondents. Similar results were observed by Zauli (2012) and Dreno (2018), who explained these gender differences by the differences in perception regarding appearance and the level of cosmetic concern. Another explanation may be the longer duration of acne in females, caused by an earlier pubertal stage.^14^, ^29^

Discrepancies were found for the influence of age on QOL. Previous studies have demonstrated a greater impact on QOL in older individuals with acne than in younger ones; however, we were not able to detect this difference. Perhaps the small number of respondents over 40 years of age was the reason.^30^

Moreover, we found that increased acne severity was associated with a more impaired QOL, and the impairment significantly differed between PtGA severity grades. However, no statistically significant differences were found between highly severe acne stages (PtGA 3 and 4). This was probably due to the small sample size of respondents representing PtGA 3 and 4, although additional analysis of both PtGA groups 3 and 4 as one group did not change these findings. These results might indicate that the magnitude of acne on a person's QOL is almost equal among PtGA grades 2, 3, and 4 (corresponding to moderate to severe acne). Our findings are consistent with several studies that found acne severity to be strongly correlated with CADI‐investigated QOL^14^, ^31^, ^32^, ^33^; however, they contradict others, which found that patients with even mild levels of acne experience a major impairment of QOL.^34^, ^35^, ^36^, ^37^ The trend toward differences in QOL at higher PtGA severity scores were, to our knowledge, new findings in this research field.

A strength of this study is the use of a validated CADI questionnaire, which is age‐appropriate and acne‐specific.^4^, ^20^ Moreover, acne severity was patient‐reported, rather than clinician‐evaluated. This might call into question the accuracy of the acne severity assessment. However, the use of the validated PtGA acne severity scale provided respondents with the opportunity to visually assess and describe their acne in a standardized manner.^22^

There were also several limitations to the study. First, the study sufficiently demonstrated a broad diversity in gender, age, educational level, geographic location, and caregivers attended, contributing to a representative sample of the Dutch acne population. However, the sample size of 384 was not reached. This might have led to the lack of detection of a significant difference between caregivers. Secondly, information about the Global QOL improvement was solicited through our own self‐developed question. This may call into question the construct validity and therefore interpretation with cause is recommended for this topic. Furthermore, the Global QOL improvement score provided valuable information that made it possible to discern the differences in QOL before and after care. However, these differences were obtained by retrospective patient perceptions of their QOL before and after care, at one moment in time, rather than through pre‐ and post‐care measurement. This method may have introduced recall bias. It is therefore recommended to further investigate the impact of the treatment on QOL in a prospective manner. Moreover, as we included in our study only individuals with acne, we were constrained to comparing QOL scores to predetermined “optimal” QOL scores in the general population. It is therefore highly recommended to establish an optimal QOL as an endpoint for caregivers to work toward in their clinical practice and to fulfill the unmet needs of individuals with acne. In addition, although we detected small differences in the QOL scores between caregivers, the CADI questionnaire does not recognize a minimal clinically important difference, to reflect changes in the QOL scores that are meaningful for the patient (such as the Dermatology Life Quality Index and the Acne‐Specific QOL Questionnaire).^38^, ^39^ It is therefore recommended to establish a minimal clinically important difference for CADI to detect differences in QOL scores that provide a more accurate interpretation of the results.

In conclusion, this study delineated factors that influence QOL in individuals with acne. As the QOL was not associated with the type of caregiver, the QOL before and after professional care, which was investigated between different combinations of caregivers, demonstrated a statistically significant improvement when attending a dermatologist, compared to the care provided by beauticians. Females and individuals dealing with more severe types of acne experienced a more impaired acne‐related QOL. Based on these results, it is recommended to take these factors into account in acne management to optimize professional treatment in line with patient needs. Identifying the possible value of care(givers) on the acne‐related QOL may contribute to adopting QOL measures as an integral part of acne management. This may improve the quality of acne care provision in clinical practice, that is more in line with the patients' needs.

## DATA INTEGRITY

Femke de Vries has full access to all of the data in this study and takes complete responsibility for the integrity of the data and the accuracy of the data analysis.

## CONFLICT OF INTEREST

The authors have no conflicts of interest to declare.

## AUTHOR CONTRIBUTIONS

Conceptualization: Femke de Vries, Esther Tjin, Rieke Driessen, Hans Vehof, Peter van de Kerkhof

Data Curation: Femke de Vries, Anissa Westenberg

Formal Analysis: Femke de Vries, Anissa Westenberg, Hans Vehof

Funding Acquisition: Femke de Vries, Esther Tjin, Peter van de Kerkhof

Investigation: Femke de Vries, Esther Tjin, Rieke Driessen, Peter van de Kerkhof

Methodology: Femke de Vries, Esther Tjin, Rieke Driessen, Hans Vehof, Peter van de Kerkhof

Project Administration: Femke de Vries, Peter van de Kerkhof

Resources: Femke de Vries, Esther Tjin, Rieke Driessen, Anissa Westenberg, Hans Vehof

Software: Femke de Vries

Supervision: Femke de Vries, Esther Tjin, Rieke Driessen, Peter van de Kerkhof

Validation: Femke de Vries, Esther Tjin, Rieke Driessen, Anissa Westenberg, Hans Vehof, Peter van de Kerkhof

Visualization: Femke de Vries, Esther Tjin, Rieke Driessen, Anissa Westenberg, Hans Vehof

Writing – Original Draft Preparation: Femke de Vries

Writing – Review & Editing: Femke de Vries, Esther Tjin, Rieke Driessen, Anissa Westenberg, Hans Vehof, Peter van de Kerkhof

All authors have read and agreed to the final version of the manuscript.

## TRANSPARENCY STATEMENT

The authors affirm that this manuscript is an honest, accurate, and transparent account of the study being reported; that no important aspects of the study have been omitted; and that any discrepancies from the study as planned (and, if relevant, registered) have been explained.

## Data Availability

The data that support the findings of this study are available from the corresponding author upon reasonable request.
